# Book Reviews

**Published:** 1814-04

**Authors:** 


### CRITICAL ANALYSIS

### OF RECENT PUBLICATIONS

IN THE

### DIFFERENT BRANCHES OF PHYSIC, SURGERY, AND MEDICAL PHILOSOPHY.

*An Essay on Medical Economy, comprising a Sketch of the State of the Profession in England, and the Outlines of a Plan calculated to give to the Medical Body in general an increase of Usefulness and Respectability.* 8vo. pp. 160. Underwood, 1814.

IT has fallen to our lot, of late years, to hear much of medical reform. The public, indeed, and with great reason, seem well contented with the skill, the talent, the integrity, and the general conduct of the various practitioners whom they patronize. The profession is divided and subdivided, until there is a distinct practitioner for every organ; one attends to the eye, another to the ear, a third to cleaning and making teeth, a fourth to extracting them; we have one practitioner for the liver, another for the heart, the lungs, the bladder, the urethra; in short, the division of labour in the medical art has become so minute, that the physician who undertakes to cure all complaints, is left with very few patients. The apothecaries abuse the druggist; surgeons the physician; the latter complains of quackery; but in reality is circumscribed in every direction.—The natural consequence of all this, is the generation of discontent and jealousy; plans of reform necessarily originate amongst those who have leisure to devise them, and who at the same time feel the necessity of something being done to improve their own condition. Now, in our opinion, this state of things by no means proves the want of a reform, or that the medical profession is degraded, or that its members are less competent to perform their duties than were their ancestors, although some of them will never be surpassed in fame, honour, or worth. The difficulty lies within a small compass, and the evil must eventually cease by a natural and obvious consequence. To use the language of political economy, the market is overstocked, and those dealers who keep up the price of their commodities will find fewer purchasers, or those who cannot afford to wait, must quit the ground. An act of legislature may entitle men to practise, but it cannot effect what is alone accomplished by nature and art, by the operation of education upon a finely organized frame; neither can it oblige patients to resort to any particular class of practitioners. This brings us immediately to the subject of the present volume, which is dedicated to Sir James Mackintosh, M. P.—We know nothing of the author, who has chosen to conceal his name; but whoever he be, he is a man of talent, research, and observation; thinks for himself, and writes in a deliberate masterly style, much above the common class of medical writers. Were it not, then, that we have often seen the well-known adage exemplified, *Nihil tam absurde dici potest, quod non dicatur ab aliquo philosophorum*, we should be somewhat surprised that a writer thus gifted, should have planned a scheme so Utopian, so impracticable, so contrary to the principles of political economy, as that which we are about to communicate. It has indeed been well observed of philosophy, that it has *tant de visages et de varieté, et a tant dit, que tous nos songes et reveries s'y trouvent.*

The author commences with an attempt to prove that medicine has not advanced in an equal degree with other arts, and reduces the causes of this imperfection to two. The first he very properly attributes to the complicated nature of the art itself; medicine, he observes, being founded on a knowledge of the laws which regulate the phenomena of organized or living bodies, while all the other arts of life spring from an acquaintance with the laws of the phenomena observed in inanimate matter.

"Now the phenomena exhibited by living bodies are not only more intricate than any other, but before a rational attempt can be made to investigate them, all that is known concerning dead matter must be rendered familiar to the mind. To connect causes and effects in medical inquiries, or to obtain logical conclusions, with regard either to the operation of remedies or the origin of disease, is hence a more arduous task than almost any other to which the mental powers can be applied; its accomplishment demands at once the widest range of acquired knowledge, the utmost patience and nicety in observation, and the most profound sagacity in deduction."

In this view of the case we fully concur, and think it quite satisfactory; but we cannot admit the second cause to be just or true; the author contends that it is owing to want of ability lity in the professors of the art, that it suffers; and that men of talents have not sufficient inducement to continue in the profession. Bad as the profession is in point of emolument, we utterly deny any destitution of ability on the part of practitioners; and so high is the encouragement for their exertions, that we find a very moderate share of talent will enable a man to rise to enviable distinction. This the author would attribute to the arts of address; we agree with him, that these too are very essential; but we also may observe that real talent will make itself conspicuous without the aid of varnish, though we cannot perceive why a man of science should deem it necessary or essential, to assume the manner and appearance of a clown, in a sick chamber, where decision and firmness of character should always blend with delicacy and refinement.

We have said thus much at setting out, because we conceive that having admitted the first cause to be valid, but at the same time irremediable in the present state of things; and shewn that the second is not founded on truth, the necessity for a reform, on the author's principles, does not exist; consequently his plan, even if feasible, is useless. Before submitting it to our notice, he takes a view of the medical establishment as regulated by law and its defects. Amongst these we were particularly amused with a complaint of "the high standard price put by physicians on their labour;" the author is quite indignant that a guinea should be claimed for every visit or prescription; yet a little before, he lamented that men of talents had no encouragement to continue in the profession. His second objection is equally frivolous, "the equality of rank among the members of the medical body, and the consequent equality of their standard fees." The author writes too well for us to suppose for a moment, that he sincerely thinks that the day on which a fellow or licentiate is admitted at the College he becomes equal in rank to his seniors, or that his fame and reputation are established with the public by passing an examination. The amount of his fees will presently convince him that his rank is merely nominal; and if by ability, address, or good fortune, he succeeds in acquiring practice at an early period, which is sometimes the case, it obviates another silly observation of the author, that "equality of rank is also objectionable, as it checks that ardour of pursuit and thirst of distinction which characterizes the juvenile mind." On the contrary there is every excitement to ardour, every encouragement to honourable and liberal exertion. The field is open to all, and the nominal rank merely entitles candidates to press forward with every fair endeavour for public approbation.

The third objection, which the author thinks is of less moment than those just mentioned, is "the restriction of the honours of the College, together with the management of the affairs of the profession, to graduates of Oxford and Cambridge." This we have ever considered a great evil, and an unjust monopoly, favouring the two English Universities at the expense of all others; encouraging those intended for physicians to become graduates of Oxford and Cambridge, where they cannot obtain an adequate knowledge of their profession, and excluding graduates of the first medical schools in Europe. To obviate the inconveniences consequent upon this system, and to prevent the universal outcry which would go forth against this grievous abuse, the College, it is well-known, have permitted a certain class of physicians, or *alieni homines*, as they have been quaintly termed by a learned *fellow*, upon payment of a sum of money, to practise their profession in London and its vicinity! So humble have the licentiates become, that they now endure their hard condition without even uttering a murmur of discontent. The time has arrived when the privileges of physicians are trampled on by all descriptions of practitioners, and the licentiates have the additional disgrace of not being allowed to defend themselves, for they have no voice or influence whatever in the community of physic, although some of them have sustained as high a degree of reputation and fame as can be acquired in the practice of medicine. We think the author might have expatiated upon this subject, and exposed its evil tendency with more force than he has employed; certainly it merits deep attention at this juncture.

Without further delay we shall transcribe the author's sketch of his grand plan.

"1st. The practice of the healing art to be confined altogether to physicians, or at least to men who have been found on trial to possess all that general and minute information now expected in candidates for the medical degree; besides including what is considered to be wanted in the surgeon only.

"2d. The members of the medical body to be divided into orders, according to seniority, with an appropriate fee for each, and every one to practise a specified time in each of the inferior departments successively, before he can be eligible for admission into the highest.

"It will perhaps be sufficient for every useful purpose to institute three orders, and for their fees we may put down half-a-crown, a dollar, and a guinea. The first may be denominated junior, the second median, and the third senior doctors, or physicians.*

"* We introduce these terms, and put down specific sums, for the sake of giving form to the subsequent part of the inquiry, without meaning to insist on the adoption of either the one or the other. The principle of dividing the body into parts, with a division of fee proportioned to the ability of the different classes of the people, is all we contend for. It is, however, essential for the guidance of the public, that

"3d. The juniors and medians to practise in all the departments of the profession, or to take the place of the general-practitioners of the present establishment, and the seniors as physicians do now; or devoting themselves to particular departments, they might act chiefly in these as consulting physicians, surgeons, accoucheurs, &c.

"4th. After devoting the specified time, say five years, to one order, the higher may be assumed at the option of the candidates; but it must be acquired by a fresh course of trial, and not simply by length of service.

"5th. The business of the profession to be managed by a supreme college, of which every practitioner must be a member, and auxiliary associations or committees, to superintend more particularly the interests of the different branches of science connected with the art.

"6th. The admission into the college to be by a course of rigid experimental examinations, and graduates of all universities to be equally eligible to become candidates.

"7th. Physicians of the senior order to be allowed to retain a certain number of the junior, as assistants or protegées, to whom they may entrust the more laborious and least valuable part of their work, while they give in return the assistance which may be expected from an overseer or counsellor.

"8th. The business of pharmacy to be left entirely in the hands of chemists and druggists; and no compounded preparation to be sold without a prescription, having the name of the prescriber written upon it at full length. The pharmaceutist to act, of course, under a licence, and the qualifications of his shopmen to be ascertained by examinations, &c.

"9th. The sum to be paid for every prescription to be marked on the receipt by the physician; and for this purpose, the prices to be adjusted periodically between a committee of physicians and pharmaceutists.

"10th. Lists of the different orders of physicians and of the pharmaceutists to be printed for general distribution every year, and a copy of the first to be hung up in every dispensary or drug shop."

---

that each division of practitioners should possess a distinct and significant appellation, in addition to the generic term. For the choice of such our language is singularly unhappy; even the genericterm *doctor* is objectionable, as it is common to all the learned professions, and consequently does not indicate to which the possessor belongs. The other, *physician*, is no less faulty in its derivation, (from φυσικη, natural philosophy), and to the ear it is not very pleasing as a familiar appellative. Were it desirable to introduce a new term, and in a case like the present, in which the aim should be perfection, a slight innovation might be warranted, the Latin *medicus* would probably be first suggested. It is, however, unsuitable, as we could only naturalize it in the singular number. From this objection the Italian word *medico-medicoes* is free; and if once familiarized to our ears, would sound, there is little doubt, as well as the title of doctor. I should have adopted this term in the text, but that I am more anxious about the establishment of principles than words, and unwilling to disturb the prejudices of those who plume themselves on verbal honours."

A con-

A considerable portion of the volume is occupied in illustrating and supporting this outline: we have only time and space to offer a few general remarks upon the subject.

The leading feature of the plan is to convert apothecaries into physicians, to divide these into three classes, and to reconcile the public to the change, by reducing the fees to half-a-crown and a dollar, for the first and second, for a limited series of years, and giving a guinea only to the third. If the author supposes that by degrading the class of physicians, which must inevitably be the case if his plan should operate, the profession would be improved, he has the experience of all ages against him. Is it nothing to reduce to one level every class of talent, connections, and industry? Is it nothing to fetter every generous youth upon starting in life with a sweeping regulation that would bring all his acquirements to one common standard? Is it possible for a moment to imagine that a man of family, fortune, and education, will ever submit to the severe and mortifying probation to which this quixotic plan would compel him? Certainly not.—In the present condition of things, the three grades, of physicians, surgeons, and apothecaries, are adequate to every good purpose. The candidate for success in either department knows the difficulties in each; but he is not cramped by any drawback that would benumb his exertions, and render useless the finest talent, address, industry, and merit. The man who does not care to risk these higher qualifications, has still the option of commencing life in the third rank, where he is morally certain, if properly qualified, of getting more emolument, than would be the case if he were a physician, *even with two-and-six-penny fees.*

---

*Official Papers relating to Operations performed by order of the Directors of the Royal Hospital for Seamen at Greenwich, on several of the Pensioners belonging thereto, for the purpose of ascertaining the general Efficacy of the New Modes of Treatment practised by* Mr. Adams, *for the Cure of the Various Species of Cataract, and the Egyptian Ophthalmia.* Published by Order of the Directors.

As much speculation has been excited by Mr. Adams's operations for Cataract in Greenwich Hospital, and his treatment of the Egyptian Ophthalmia, we have great pleasure in transcribing the following extracts from the Official Papers just published.

"In the autumn of 1812, the directors of Greenwich Hospital were informed that great improvements had been recently made by Mr. Adams, late Oculist to the West of England Eye Infirmary at Exeter, in the modes of operating on the different kinds of cataract; and and as the practice of extraction heretofore performed on the pensioners had not been attended with the desired success, he was requested to examine the blind pensioners, and accordingly reported, that, 'with the surgeon of the hospital, he had selected twenty cases for operation, consisting of cataracts, closed pupils, and the Egyptian Ophthalmia; and as on the two former diseases he had recently published some important discoveries, and also successfully adopted a novel mode of practice in the worst stages of Ophthalmia, he entertained a confident expectation that the superior efficacy of his operations over those usually practised would enable him, in some of the most formidable diseases of the eye, to afford relief to many of the pensioners, whose cases had been considered incurable; but he utterly disclaimed all expectation of remuneration for his professional services on this occasion.'

"The pensioners being desirous to be placed under the care of Mr. Adams, the medical officers of the hospital were directed to attend the operations, to afford every assistance, and to report to the directors the progress and result. A house was accordingly taken in Air-street, Piccadilly, for the accommodation of the pensioners, and the convenience of the oculist; but being found, in many respects, unsuitable, another was afterwards hired in Lisle-street, Leicester-square, every way adapted to the comfort of the patients.

"It appearing, by the reports of the medical officers of the hospital, that the operations performed on the blind pensioners by Mr. Adams, had been attended with great success, the directors were desirous of viewing the men, and for that purpose convened a special meeting, at which the pensioners, as well those who had been under the care of former oculists, as the patients of Mr. Adams, were examined and individually interrogated; and as the effect of the operations performed by the latter, as stated in the reports of the physician, surgeon, and apothecary, was found to be accurately detailed, the directors have considered it to be their duty to give publicity to the official reports and proceedings on a subject so interesting to humanity.

"OFFICIAL PAPERS, &c.

"(COPY.) "*May* 25*th*, 1813.

"Sir,—Conceiving that it will be gratifying to the governor and directors of the Royal Hospital to hear that the new practice for the cure of cataract, and the formation of artificial pupil, proceeds most favourably on the pensioners placed under the care of Mr. Adams, I have great pleasure in acquainting you, for their information, that the first set of patients sent to London, requiring thirteen eyes to be operated on, are all cured, with the exception of one man, David Hoar, a person of notoriously perverse character, who was attacked with fever, and sent back to the hospital for the cure thereof; but even in this case there is every prospect of success, from a repetition of the operation. Eleven other patients who were sent to re-place those already cured, have all been operated on, and with the prospect of complete success (excepting two that had been previously couched by another oculist), five being already capable of seeing, and the others proceeding as favourably as could be expected.

"The superior success of Mr. Adams's new modes of practice, when compared with the operation performed on the pensioners for the last fifteen years, is very striking. On examining the latter it appears, that out of *twenty-four eyes,* operated upon, several had been destroyed; in other instances the pupils had become obliterated, and *one only* had been benefited, and even in that the success is incomplete.

"Among the men already cured, some of the cases are so remarkable as to merit particular notice.

"Edward Turner had, during six months that he was a patient of the London Eye Infirmary, undergone *thirteen* operations; nevertheless, he obtained but very little benefit in one eye, and none in the other; Mr. Adams has cured both by *one operation on each.*

"Hartgill had been blind for near twenty years, and was considered by every oculist of eminence in London to labour under gutta serena. Mr. Adams has successfully operated on him, and he is now capable of reading the *smallest print.*

"Bray, aged seventy-nine years, is cured of cataract in both eyes, and was capable, in the space of a fortnight, of seeing the *minutest objects.*

"Douglas, aged thirty-two, with closed pupils, after having been above five years a patient of the oculist before referred to, without experiencing the least benefit, has had an artificial pupil formed, by which he is enabled to see the most minute objects with distinctness; his other eye has been since operated on, and promises to be equally successful.

*John Dyer, Esq. Surgeon.* B. M'LAUGHLIN, *Secretary.*

*Royal Infirmary, Dec.* 27, 1813.

"Sir,—We enclose herewith, for the information of the directors, separate reports of the results of the *new* and *old* operations for the cure of cataract which have been practised upon the pensioners of the Royal Hospital, the great disparity in which cannot fail to make a strong impression on the minds of the honourable members of the board.

"In order to bring them equally acquainted with the extent of the failures in extracting the cataract, as formerly practised, as well as with the great success of Mr. Adams's improved modes of curing that disease, we have given a detailed description of the result of each, with the present state of the eyes which have been submitted to the trial of the two systems.

"The proportion of eyes totally destroyed by the operation of extraction amounts to one-half the number operated upon; to this the success of Mr. Adams, more particularly in the cases which had been considered incurable, as well as those previously operated upon without benefit, forms a very striking contrast, as it will be seen that his operations have failed but in one instance.

"To enable the board fully to appreciate this success, we think it proper to point out, that even in the men whose vision is not at all, or but partially, benefited (with the exception of Ford), the operations were as perfectly executed as on those whose sight is completely restored. To the disease of the optic nerve, therefore, and not to the failure of the operation (as was the case where extraction had been formerly formerly performed), is to be attributed the want of that perfectly successful issue which is so conspicuously displayed in the '*unexceptionable cases.*'

"This diseased state of the optic nerve in those patients was originally apprehended by Mr. Adams; and when, at their urgent solicitations, he was prevailed upon to perform the necessary operations, he stipulated, that, should the event confirm his unfavourable opinions, we should attest the circumstances under which they were undertaken.

"It is, however, very important to have ascertained, by actual experiment, as Mr. Adams has done on several of the pensioners, that the optic nerve, although so much diseased as to have deterred a former practitioner from operating, yet, by the removal of the cataracts, and subjecting the eyes to a particular plan of discipline, their functions have been sufficiently recovered to afford useful, and sometimes almost perfect, vision. An instance of the latter is shewn in the case of Hartgill, blind for nearly twenty years, as supposed, by all the highest authorities in London, from gutta serena, for which disease he had been treated. Bray's and Wilkins's perfect restoration to sight are little less extraordinary, from the great age of the former, and the latter having had an artificial pupil formed after a complete obliteration of that aperture, by an unsuccessful operation of extracting the cataract, performed seven years since.

"These, together with the other cases included in the two last divisions of the Report No. 2, prove that a very large proportion of persons unhappily afflicted with blindness, and hitherto considered incurable, are now susceptible of relief from the new and improved operations, and the after-management of the eyes, practised by Mr. Adams.

"In addition to the gratifying contents of the second Report, we think it our duty to state, for the information of the board, that Mr. Adams has discovered a mode of curing the Egyptian Ophthalmia, which has been successfully practised upon several of the pensioners, some of whom had been blind for three or four years, and given up as incurable by the most eminent oculists then in London. The communication that this destructive and hitherto intractable disease admits of cure we conceive will be gladly received by the board, and the promulgation by Mr. Adams of this important discovery be considered as a great *national desideratum.*

"By the adoption of his practice we are of opinion, from what we have seen of its effects, that a very large proportion of the seamen and soldiers, who have been discharged the service, blind of the Ophthalmia, might be again rendered fit for duty, or be made useful members of society.

"We cannot conclude this letter without stating, in justice to Mr. Adams, that he has freely demonstrated his practice; and that he has, in the most liberal and unreserved manner, given us every information that we required relating to the treatment of diseases of the eyes. We are, Sir, your very humble servants, R. ROBERTSON, *Physician.*

"B. M'LAUGHLIN, *Surgeon.*

"*John Dyer, Esq.* "M. S. KENT, *Apothecary.*

"RESULT of the OPERATION of EXTRACTION which had been performed on Pensioners blind of Cataract now in the Hospital, previous to the employment of Mr. ADAMS.

| | | |
|---|---|---|
| REPORT 1. | Eyes destroyed | 12 |
| | Obliterated Pupils | 4 |
| | Gutta Serena and secondary Cataract | 3 |
| | Opaque Cornea, and other diseased changes of the Eye | 4 |
| | Successful | 1 |
| | Total number of Eyes upon which the operation of Extraction had been performed | 24 |

RESULT of the NEW OPERATIONS performed on the Pensioners by Mr. ADAMS.

CASES considered unexceptionable.

| REPORT 2. | Age. | Description of Disease. | Number of Eyes operated upon. | Result of the Operations. | State of Vision. |
|---|---|---|---|---|---|
| John Bray | 80 | Cataract | 2 | Perfect | Perfect, being able to see as well as ever with Cataract Spectacles. |
| Richard Collins | 63 | Do. | 2 | Do. | Perfect, do. do. |
| Robert Kinsley | 70 | Do. | 2 | Do. | Sight returning, when he died from Carbuncle. |
| Robert Handcock | 47 | Do. | 1 | Do. | Perfect, and gone on Out-Pension. |
| *Jonathan Stratton | 75 | Do. | 1 | Do. | Perfect. |
| Edward Hilback | 40 | Do. | 1 | Do. | Do. |
| Guy Overton | 79 | Cataract, with obliterated Pupil | 1 | Cataract cured, and an Artificial Pupil formed | Do. |
| Silas Darby | 66 | Cataract | 1 | Perfect | Do. |
| William Russell | 50 | Cataract complicated, with obliterated Pupil, and a defect of the Optic Nerve | 1 | Cataract cured, and an Artificial Pupil formed | Vision nearly, but not quite perfect, owing to the diseased state of the Optic Nerve. |
| William Roberts | 48 | Cataract | 1 | Cataract cured, partial opacity of the Cornea remaining | Vision not perfect, but sufficient for the common purpose of life. |
| Thomas Ford | 74 | Do. | 1 | Unsuccessful, but no deformity produced | |

CASES considered incurable by former Oculists.

| | | | | | |
|---|---|---|---|---|---|
| John Douglas .... | 36 | Cataracts with obliterated Pupils .... | 2 | Cataracts cured, and Artificial Pupils formed ................ | Perfect. |
| Frederick Hartgill | 48 | Cataracts complicated, with Gutta Serena .................... | 2 | Perfect ........................... | Was enabled to read small print with great fluency, till attacked with violent inflammation of the eyes from cold. Is now convalescent. |
| †Thomas Dailey .. | 46 | Cataract and obliterated Pupil, with Gutta Serena ............. | 1 | Cataract cured, and an Artificial Pupil formed ..................... | Vision useful, and can see small objects. |
| William Austin .. | 31 | Do. Do. Do. | 1 | Ditto .................... | Vision useful, and able to see objects at a considerable distance. |
| David Hall ...... | 52 | Cataract and obliterated Pupil ........ | 1 | Convalescent when discharged from the Infirmary for incorrigible drunkenness ................ ... | |
| William Thompson | 49 | Do. Do. ............ | 1 | Cataract cured, but Pupil requires further enlargement .......... | Vision useful and improving, when he died of consumption. |
| †Otter Grindall .. | 40 | Cataract and Gutta Serena .......... | 1 | Perfect ........................ | Vision useful, and continues to improve. |
| Thomas Whiteman | 28 | Cataracts (adherent) with a defect of the Optic Nerve .......... | 2 | Do. ........................ | Vision very good, but not perfect. |

CASES which had been operated upon unsuccessfully by former Oculists.

| | | | | | |
|---|---|---|---|---|---|
| Edward Turner .. | 46 | One cataract adherent, the other detached & floating. Had previously undergone *thirteen* operations .. | 2 | Perfect ........................ | Perfect. |
| John Broadway .. | 51 | Cataracts membranous (or secondary,) and Gutta Serena .. | 2 | Do. ........................ | Not improved, Optic Nerve being totally insensible. |
| John Maddin .... | 52 | Do. do. with obliterated Pupil .... | 1 | Membranous Cataract removed, and an Artificial Pupil formed | Do. |
| John Wilkins .... | 52 | Cataract secondary, with obliterated Pupil, from the failure of Extraction seven years since .. | 1 | The secondary Cataract removed, and an Artificial Pupil formed ...... | Perfect, being able to see the minutest objects. |
| | | Total number of Eyes upon which the new Operations have been performed | 31 | | |

RECAPITULATION.

| | |
|---|---|
| Successful ................. | 29 |
| Unsuccessful .............. | 1 |
| Discharged for irregularity .. | 1 |
| Total .. | 31 |

R. ROBERTSON, *Phys.* B. M'LAUGHLIN, *Surg.* M. S. KENT, *Apoth.*

* Jonathan Stratton was attacked, three months after he was cured, with repeated violent inflammations of the Eye, which assumed an intermittent form, and has nearly destroyed the Vision.

† Otter Grindall is since dead of Apoplexy.

"26, *Albemarle-street, Jan.* 9, 1814.

"My Lords and Gentlemen,—The favorable termination of the trial which you directed to be made, in order to ascertain the comparative success of my new modes of operating for the cure of cataract, with that of the operation of extraction, as it is generally performed, will, I hope, be thought to justify my addressing you on the circumstances of it.

"Although fully aware of the dangers attending the operation of extraction, as usually performed, and apprized, as I was, that the pensioners could no longer be prevailed upon to submit to that mode of operating, from its ill success for the last fifteen or twenty years, I did not conceive, till I perused the reports of the surgeon of the hospital, that the proportion of failures was so great.

"From the statements which have been made of the success of the practice of extraction, the public have been taught to believe that it possessed all the excellence of which any operation for the cure of cataract was susceptible. It became, therefore, highly necessary that such an experiment as the present should be instituted; and that, under the immediate superintendance of *impartial* and *disinterested* persons, whose testimony could not be doubted.

"It is then with no common satisfaction that I now request your attention to the comparative results of the differents operations (the *new* and *old*), as specified in the official reports of the physician, surgeon, and apothecary, to your institution; which, with the personal examination you intend this day to make of the two sets of patients, must necessarily establish, beyond all doubt, the decided superiority of my modes of operating, over that which had been previously practised on the pensioners.

"And here I beg leave to repeat the observation I made at my first interview with your honourable board, that it is the *operation*, and not the *operator*, which I deprecate. Were he to adopt my operations, or were I to follow his, the results of the two modes of practice would probably be nearly the same as they are now found to be; nor shall I hesitate to add my firm belief, that superior manual dexterity is not to be found in this kingdom, than is possessed by the operator whose efforts have proved so unavailing, in the many instances submitted to your consideration. It is, I conceive, the want of a personal experience of the superior efficacy of my practice, which prevents his adopting it with the same promptitude, as another oculist of long-established celebrity has done, since he saw me operate; who, before that period, was distinguished by his practice, as well as writings, as one of the warmest advocates of the operation of extraction.

"It may be proper to inform your honourable board that I have not confined myself to any individual operation in the treatment of the pensioners blind of cataract intrusted to my care. My instruments and modes of operating have varied as the nature of the case required. Where the consistence of the cataract has admitted of an immediate and complete division, I have placed the separated portions in a situation which ensured their absorption in five or six weeks. In these cases, the general success of the operation exceeds all credibility with those who have been in the habit of witnessing the results of other modes modes of practice. Of upwards of *eighty persons* born blind of cataracts, upon whom I have performed this operation, I have not lost an eye. In three instances alone, in which I was prevented from repeating the operation, it did not produce the anticipated benefit; and I should consider myself unfortunate were I at any time to be less successful in an equal number of persons who became blind from cataracts after birth, provided they admitted of being treated in the manner already described, and the health of the patients was in a state favourable for the operation. Hence arises a very important question: To what period of life does this particular practice apply? To which I have a ready answer: That I have *never failed* in being enabled to effect this necessary division in persons under *forty years* of age; very rarely in those between *forty* and *fifty*, and have frequently succeeded in persons in the most advanced periods of life.

"Where the cataract is too hard and solid to admit of this immediate division, I do not attempt, as was my former practice, to effect its absorption by a frequent repetition of the operation; but I at once extract it. This, however, is accomplished by a process *totally different* from that I have felt it a duty to deprecate; a process which I must claim to be novel, and which happily attains the highly important desiderata which had been hitherto considered *unattainable*, while it obviates the many causes of failure which rendered the usual mode of extraction so generally unsuccessful. From the principle upon which it is founded, and the favourable results of its termination during the last two years that I have extensively practised it, I feel myself warranted in asserting that it possesses the utmost degree of excellence which it is *possible* for extraction to arrive at, and that its general success will prove nearly as great as the operation for the removal of the soft cataract. To deter other persons from claiming it as their invention, or anticipating me in its communication to the public (as was the case with my instruments and operation for the cure of the *soft cataract*, and my successful revival of an obsolete operation for *artificial pupil*), I have requested Mr. M'Laughlin to record on the hospital books, the different stages of this operation, as he has seen me perform it on several of the pensioners.

"In many cases an artificial pupil has been made, as well as the cure effected of the cataract, with which the disease of obliterated pupil was complicated. Again, where the pupil, though not obliterated, was much contracted from adherent cataract, a different operation was practised. In other instances I have removed secondary or membranous cataracts, which had come on after the usual mode of extraction had been to all appearance perfectly accomplished.

"I have also in my treatment of the pensioners ascertained a fact of very great practical importance, which will in a great degree explain the general bad success of the operation of extraction, as it is usually performed; namely, that the vitreous humour was in a state of dissolution nearly in one half of the eyes on which I operated. This is a diseased change which can rarely be perceived before the performance of the operation, and which authors agree must occasion a total destruction of the eye, whensoever the cataract is extracted in the usual manner. In these cases I performed an operation of a different kind from any of the former.

"The

"The happy result which has attended such a combination of practice, (by which peculiar and appropriate instruments and modes of operation have been adapted to each variety of the disease) proves that those who pursue one beaten track, in all cases, must necessarily fail in a very large proportion of them; and still further accounts for the bad success formerly attending the operation of extraction on the pensioners.

"I trust that it will not be considered as irrelevant to the subject of the present communication to inform you, that there are different modes of effecting the cure of cataract by the *absorbent practice.* My friend and preceptor, the late Mr. Saunders, pursued a system different from that which I have so warmly supported in this letter. The operation which he preferred had been performed *thirteen times* during six months on one of the pensioners (Edward Turner) without a removal of the disease. On one of my private patients the same operation had been performed *seventeen* times prior to my having been consulted, *ten* times on one eye, and *seven* on the other, in the course of as many months, and with no better success. In both instances I perfected the cure by a single operation on each eye; so that, if these patients had originally been treated according to my mode of practice, *one*, or, at most, two operations, would have effected the complete removal of the cataracts in the space of five or six weeks. This difference in the two modes of operating, it is of great importance to myself distinctly to specify, otherwise, from its being generally known that I was the sole confidential pupil and assistant of the late Mr. Saunders in his operations for cataract, among those who are now pursuing the profession of an oculist, it might be considered by many, who have not seen my work on Diseases of the Eye, that I still, as in the commencement of my practice, follow his modes of operation, whereas I have long since found it necessary wholly to abandon them.

"I must now, my lords and gentlemen, beg leave to apologize for occupying so much of your time and attention; but I trust that you will attribute the length of my present communication to an anxious desire to give you some explanation of the nature of those operations, by which I have been enabled so successfully to fulfil your wishes in the treatment of the pensioners.

"While I offer my most grateful acknowledgments for the politeness and condescension with which, collectively and individually, you have been pleased to receive my communications, and to acquiesce in those arrangements which I presumed to recommend for the better trial of the important experiment just decided,—permit me also to express my warmest obligations to the medical officers of your institution, for their liberal and zealous co-operation. In an especial manner it becomes me to mention the humane and able assistance afforded by Mr. M'Laughlin, to which may be attributed much of the successful issue of my experiments.

"I have the honor to remain,

"With the highest respect,

"My Lords and Gentlemen,

*To the* "Your most obedient humble Servant,

*Hon. the Directors of Greenwich Hospital.* "WILLIAM ADAMS."

"*At*

"*At a Meeting of the Directors of Greenwich Hospital, at that Place, on Monday, the* 10*th of January,* 1814,

PRESENT,

CAPTAIN BROWELL, *Lieutenant-Governor,*

| | |
|---|---|
| LORD AUCKLAND, | MR. YENN, |
| Reverend MR. COOKE, | DR. ROBERTSON, |

"The Board resumed the consideration of the letter of the 27th of last month, from the physician, surgeon, and apothecary, and of the report which accompanied it, detailing the effect of the operations performed by Mr. Adams on several of the pensioners afflicted with cataracts, and other diseases of the eyes; and the physician being at the Board, Mr. Adams, and also the apothecary and surgeon's two assistants (the surgeon being absent by indisposition), were called in; and the Board, with a view of ascertaining the comparative success of the different modes of practice, in cases of cataract and closed pupil, proceeded to examine the pensioners on whom operations had been performed by other oculists of undoubted character and eminence in their practice. It appeared that many of these pensioners had irrecoverably lost their sight, the eye being in several instances entirely sunk; and that, except in one instance, they had not experienced the desired relief.

"The pensioners who had been under the care of Mr. Adams were next examined and interrogated, and their respective cases were compared with the report enclosed in the above-mentioned letter from the physician, surgeon, and apothecary; and the Board were much gratified by so many instances of the great success which had attended the operations of Mr. Adams. The effect of those operations appeared to be accurately detailed in the report in question.

"Mr. Adams then laid before the Board a letter of this date, stating at considerable length his modes of practice; and he also personally explained the nature and effects of the several operations performed by him for cataract, and in forming an artificial pupil. In the result the Board expressed to Mr. Adams their entire satisfaction, and requested he would select such other of the blind pensioners belonging to the Hospital whose cases may be treated with any prospect of success; and that he would perform such operations on them as in his judgment may be calculated to afford relief; and then, together with the medical officers, he withdrew.

"It being evidently desirable that publicity should be given to the success which has attended Mr. Adams's operations in restoring to sight so many pensioners of the Hospital:—

"Ordered,

"That the letter and the report above-mentioned from the medical officers of the Hospital, together with Mr. Adams's letter of this date, the surgeon's two letters of the 26th of May and 28th of August last, and the proceedings of the Board relative to this subject, be forthwith printed and published."

*A Practical Treatise on Cataract.* By JOHN STEVENSON, Oculist and Aurist to her Royal Highness the Princess Charlotte of Wales, Member of the Royal College of Surgeons, &c. The second Edition, with considerable additions; 8vo. pp. 224. Longman and Co. 1814.

As we so lately noticed the first edition of this work, we have little to remark upon the present occasion. The author has entered more into detail, and introduced some new pathological observations, and added several cases which his increased experience has enabled him to supply.

The following remarks in the preface apologize for his not inserting colored plates, which much increase the price of a book, and though they may gratify curiosity, certainly cannot form an operator.

"It was my intention, (he observes) as it has been the custom of late, to annex some colored plates. Both the artist and the practitioner must, however, be aware of the difficulty, not to say the impossibility, of successfully illustrating, in this way, the diseases of the eye. Every engraving, as it appeared well finished and interesting, or, to use the language of the artist, in proportion as it preserved the effect, I found to deviate from a just representation of the object.

"The parts of the eye are so intricate, the optical illusions so great, that professors, even in teaching the theory of vision, are obliged to use models of detached parts of the organ; and with the assistance of such demonstrations, and their own verbal explanations, find it impracticable to make themselves understood, without the closest application on the part of the student.

"The structure of the eye, in its natural state, should be perfectly familiar to every one who wishes to study, with effect, its complicated disorders. With such a knowledge, any morbid condition, if accurately detailed, will be readily understood: without it, all description and delineation must be equally incomprehensible.

"The same disease, in different persons, and in the same individual, at different times, varies so much in its appearance, as to baffle every effort to

'Catch ere she change the Cynthia of this minute.'

"To convey a correct idea of the successive changes which occasionally occur, even in the same case, would require an indefinite number of plates, since a single representation could communicate only a conception of one specific feature of the complaint.

"Equally difficult must it be to trace the progress of an instrument by an art, which exhibits only the act of an instant."

If the first edition of this treatise afforded a satisfactory proof of the author's attention to the subject of cataract, the present performance has yet stronger claims to superiority.

MEDICAL

